# Cyclooxygenase-2 Inhibition Blocks M2 Macrophage Differentiation and Suppresses Metastasis in Murine Breast Cancer Model

**DOI:** 10.1371/journal.pone.0063451

**Published:** 2013-05-07

**Authors:** Yi-Rang Na, Yi-Na Yoon, Da-In Son, Seung-Hyeok Seok

**Affiliations:** Department of Microbiology and Immunology, and Institute of Endemic Disease, Seoul National University College of Medicine, Seoul, South Korea; Istituto Superiore di Sanità, Italy

## Abstract

Tumor cells are often associated with abundant macrophages that resemble the alternatively activated M2 subset. Tumor-associated macrophages (TAMs) inhibit anti-tumor immune responses and promote metastasis. Cyclooxygenase-2 (COX-2) inhibition is known to prevent breast cancer metastasis. This study hypothesized that COX-2 inhibition affects TAM characteristics potentially relevant to tumor cell metastasis. We found that the specific COX-2 inhibitor, etodolac, inhibited human M2 macrophage differentiation, as determined by decreased CD14 and CD163 expressions and increased TNFα production. Several key metastasis-related mediators, such as vascular endothelial growth factor-A, vascular endothelial growth factor-C, and matrix metalloproteinase-9, were inhibited in the presence of etodolac as compared to untreated M2 macrophages. Murine bone marrow derived M2 macrophages also showed enhanced surface MHCII IA/IE and CD80, CD86 expressions together with enhanced TNFα expressions with etodolac treatment during differentiation. Using a BALB/c breast cancer model, we found that etodolac significantly reduced lung metastasis, possibly due to macrophages expressing increased IA/IE and TNFα, but decreased M2 macrophage-related genes expressions (*Ym1, TGFβ*). In conclusion, COX-2 inhibition caused loss of the M2 macrophage characteristics of TAMs and may assist prevention of breast cancer metastasis.

## Introduction

Tumor associated macrophages (TAMs) and the factors they release amplify various aspects of cancer, including anti-tumor immune suppression, stimulation of tumor progression, and formation of metastases [1]–[3]. High levels of TAMs are often correlated with bad prognosis, and several recent studies have highlighted a link between their abundance and the metastatic process [4]–[6]. Macrophage population ablation by genetic and pharmacological approaches can counter subsequent cancer development [7]–[10].

Analogous to the Th1 and Th2 dichotomy of T cell polarization, macrophages can be polarized by the microenvironment to mount specific M1 (classically activated) or M2 (alternatively activated) functional programs [11]–[13]. TAMs exhibit a predominantly M2-like phenotype [13], [14]. This preferential polarization is due to the abundance of M2 stimuli as well as the absence of M1-orienting signals in the tumor, such as IFN-γ or bacterial components. Indeed, M2-macrophages differentiate from monocytes in response to specific growth factors released by both malignant and stromal tumor compartments, including CCL2, M-CSF, vascular endothelial growth factor (VEGF) and CXCL12 (also known as SDF1) [2], [15], [16]. Macrophage polarization is also regulated by post-stimulations such as the M2a stimulants IL-4 and IL-13, M2b stimulants including immune complexes/TLR ligands, and IL-10 and glucocorticoids polarize macrophages into the M2c subtype [13], [17]. In general, the hallmarks of M2-macrophages are production of IL-10^high^, IL-12^low^, IL-1RA^high^, IL-1decoyR^high^, CCL17^high^, and CCL22^high^. In addition, M2-macrophages exhibit high expression of mannose, scavenger and galactose-type receptors, poor antigen-presenting capability, wound healing promotion, debris scavenging, angiogenesis, and tissue remodeling through high expression of VEGF, cyclooxygenase-2 (COX2), epidermal growth factor receptor (EGFR), and metalloproteinases (MMPs) [18]–[20]. In a tumor context, macrophages act as a guardian and induce anti-tumor immune responses in the early stages but in the later stages as the tumor progresses, macrophages enhance tumorigenesis and metastasis [19]. Macrophage polarization is the key step that accelerates tumor aggressiveness. However, data regarding the molecular mechanisms of macrophage polarization remain sparse.

Epidemiological studies have shown that regular intake of nonsteroidal anti-inflammatory drugs (NSAIDs) such as aspirin, the prototypic inhibitor of cyclooxygenase (COX), can reduce the risk of development of some cancers [21]. Thus far, several mechanisms by which COX-2 contributes to cancer progression have been reported, including stimulation of proliferation and inhibition of apoptosis of cancer cells, stimulation of cancer cell invasion and angiogenesis, and suppression of immune responses [22], [23]. In addition to its effects on tumor cells, COX is the key enzyme induced when macrophages are activated for synthesis of inflammatory mediators. These mediators include prostaglandin E2, prostacyclin I2, and thromboxane A2. Other tumor-related molecules, such as VEGF-A and VEGF-C, can also be induced by COX-2, as demonstrated by data that COX-2 inhibition suppresses lymph node metastasis via reduction of macrophage-mediated lymphangiogenesis [24]. These data provide evidence that COX-2 participates in macrophage polarization, but its exact role has not been elucidated.

Macrophages are related to tumor growth, metastasis, and relapse. Macrophage-mediated immune suppression is correlated with increased CD4^+^CD25^+^ regulatory T cell infiltration and reduced CD8^+^ cytotoxic T cell function. The proteinase secretion capacities of macrophages directly lead tumor cells for moving through the extracellular matrix degradation [25]. Additionally, recruitment of CD11b^+^ myeloid cells facilitates tumor regrowth after local irradiation therapy [26]. Therefore, various therapeutic applications to enhance tumor immunity have been attempted, including anti-TGFβ antibodies, anti-CCR4 antibodies, anti-CTLA4 or Programmed Death-1 (PD-1) inhibitory molecules, TLR7 or Bacillus Calmette-Guerin (BCG) boosting therapy, and IL-2 or IFNγ treatment.

To clarify the role of COX-2 inhibition in macrophage function in tumor context, we conducted human and mouse macrophage differentiation with COX-2 inhibitor, etodolac. Here we demonstrate that COX-2 inhibition blocks M-CSF-induced M2 macrophage differentiation and drives pro-inflammatory activities in human and murine macrophages. Regular etodolac intake inhibits breast cancer metastasis in relation to reducing M2 macrophage functions. Our finding suggest that COX-2 inhibition may inhibit M2 macrophage differentiation and polarization, thus suppressing tumor metastasis.

## Materials and Methods

### Ethics statement

All human blood acquisitions were approved by the Institutional Review Board of Seoul National University, Korea (SNUIBC-R120713-1). Documented written informed consents provided by Ethics Committee were obtained from all participates in this study. All animal procedures were performed according to the criteria outlined in the Guide for the Care and Use of Laboratory Animals prepared by the Institution of Animal Care and Use Committee of Seoul National University, Korea. The protocol was approved by Seoul National University Institute Animal Care and Use Committee (Approval Number SNU-10009-2). All surgical procedure was performed under zoletil (Virbac)/xylazine (Bayer) anesthesia, and all efforts were made to reduce unnecessary pain.

### Human and murine macrophage differentiation and reagents

Human peripheral blood mononuclear cells (PBMC) were isolated from buffy coats of normal donors over a Ficoll-paque PLUS (GE Healthcare) gradient, according to standard procedures. Monocytes were purified from buffy coats by magnetic cell sorting using the human monocyte isolation kit II (Miltenyi Biotec). Monocytes (>90% CD14^+^ cells) were cultured for 7 days at a density of 10^6^/ml in RPMI media (Thermo) supplemented with 10% FBS (Gibco), 10 units/ml penicillin, and 10 µg/ml streptomycin and 2 mM L-glutamine (Gibco) (hereafter termed complete media) at 37°C in a humidified atmosphere with 5% CO_2_. For differentiating human M2 macrophages, 20 ng/ml human M-CSF (Prospec) were supplemented into complete media and for M1 macrophages, 25 ng/ml human GM-CSF (Prospec) were used. Murine bone marrow cells were obtained from 7∼10 weeks of BALB/c female and differentiated into mature macrophages during 7 days in 10% L929 murine fibrosarcoma cell line culture supernatants supplemented complete media. Etodolac (Yuhan Corporation, Korea) was dissolved in DMSO and treated at 20 µM for monocyte differentiation. Where indicated, macrophages were activated for 12 hours (hrs) with LPS (100 ng/ml; *Salmonella enterica*, Sigma Aldrich) and IFNγ (25 ng/ml, Prospec). 15-deoxy-Δ12,14-PGJ_2_ in methyl acetate was obtained from Cayman Chemicals and added twice (at the indicated concentrations, 0.1–2 µM), at the start of the culture and on day 5 of macrophage differentiation.

#### 4T1 syngeneic mouse breast cancer model

The 4T1 murine breast cancer cell line was obtained from ATCC and maintained in RPMI complete media. The 4T1_GFP cell line was generated through copGFP viral particle (Santa Cruz Biotechnology) infection followed by selection in 4 µg/ml puromycin for 2 weeks. Six-week-old female BALB/c mice were purchased from Orient Bio (Korea) and maintained in pathogen-free housing. For orthotopic implantation of tumor cells, mice were anesthesized with zoletil (Virbac)/xylazine (Bayer) and a total of 10^5^ 4T1 cells (suspended in 100 µl ice cold PBS) were injected into the right inguinal mammary fat pad of seven-week old mice. Etodolac was fed at 500 ppm throughout the experimental period. Animal weight and tumor size were measured twice per week. Tumor volumes were calculated as 0.5×length×width^2^. Necropsy was performed 16 and 23 days after tumor cell implantation. After administration of anesthetic overdose, blood was collected by cardiac puncture and mice were sacrificed by cervical dislocation. Lungs were removed from each mouse, surface lung nodules and sizes which could be identified grossly were blindly measured. In the case of 4T1_GFP cells, lung images were captured using a fluorescence microscope (Leica M165FC) and GFP intensity was quantified using Image J software. Resected lung, spleen, and the primary tumor mass were processed for further examination.

### Flow cytometry

Human and murine macrophages were examined for receptor and cytokine expression levels using FACS. Flow cytometry on fixed macrophages was performed using anti-CD11b APC-eFluor 780 (eBioscience), anti-human CD14 PerCP-Cy5.5 (eBioscience), anti-human CD163 PE (eBioscience), anti-human TNFα PE-Cy7 (eBioscience), and anti-human IL-10 Alexa Fluor 647 (eBioscience). Human macrophage intracellular cytokines were accumulated with Golgiplug (BD Biosciences) during 5 hrs after 100 ng/ml LPS stimulation, scrapped, fixed and permeabilized before antibody staining. The spleen and whole primary tumor mass from mice were dissected into thin slices and incubated for 30 min with 2 mg/ml collagenase A (Sigma Aldrich) and 100 U/ml hyaluronidase (Invitrogen) at 37°C. Single cell suspensions were washed three times and frozen until examination. Flow cytometry on thawed murine cells or differentiated BMDMs was performed using anti-CD11b APC-eFluor 780 (eBioscience), anti-mouse F4/80 PE (eBioscience), anti-mouse IA/IE PE-Cy5.5 (eBioscience), anti-mouse CD80 Qdot 605 (eBioscience), anti-mouse CD86 V450 (BD), anti-murine TNFα PE-Cy7 (ebioscience), and anti-murine IL-10 APC (eBioscience) antibodies. Isotype-matched monoclonal antibodies (eBioscience) were used as negative controls. Data are represented as mean fluorescent intensity (MFI) or relative percentages to control.

### Reverse Transcription Polymerase Chain Reaction (RT-PCR)

For real-time RT-PCR, mRNA from maturated macrophages or CD11b-positive magnetic cell sorted (Miltenyi Biotec) murine spleen macrophages and tumor-associated macrophages [27] were analyzed in duplicate. cDNA was synthesized from 1 µg total RNA using Maxime RT Premix (Intronbio, Korea) [28]. The primers and probes used for PCR analyses were: human *TGFbeta* forward, 5′-CGAGCCTGAGGCCGACTAC-3′; *TGFbeta* reverse, 5′-AGATTTCGTTGTGGGTTTCCA-3′; *TGFbeta* probe (FAM), 5′-CAAGGAGGTCACCCGCGTGC-3′; human *VEGFA* forward, 5′-CATGCAGATTATGCGGATCAA-3′; human *VEGFA* reverse, 5′-TTTGTTGTGCTGTAGGAAGCTCAT-3′; human *VEGFA* probe (FAM), 5′-CCTCACCAAGGCCAGCACATAGGAGA-3′; human *MMP1* forward, 5′-TTTGATGGACCTGGAGGAAATC-3′; human *MMP1* reverse, 5′-TGAGCATCCCCTCCAATACC-3′; human *MMP1* probe (FAM), 5′-TGCTCATGCTTTTCAACCAGGCCC-3′; human *MMP9* forward, 5′-CGCCAGTCCACCCTTGTG-3′; human *MMP9* reverse, 5′-CAGCTGCCTGTCGGTGAGA-3′; human *MMP9* probe (FAM), 5′-TCTTCCCTGGAGACCTGAGAACCA-3′; human *MMP2* forward, 5′-GCACCCATTTACACCTACACCAA-3′; human *MMP2* reverse, 5′-GAGCTCCTGAATGCCCTTGA-3′; human *MMP11* forward, 5′-TGCCCGACCCATCTGATG-3′; human *MMP11* reverse, 5′-CGCCAGAAAGCACGAACCT-3′; human *MMP13* forward, 5′-ATTAAGGAGCATGGCGACTTCT-3′; human *MMP13* reverse, 5′-CCCAGGAGGAAAAGCATGAG-3′; murine *VEGFA* forward, 5′-CATCTTCAAGCCGTCCTGTGT-3′; murine *VEGFA* reverse, 5′-CAGGGCTTCATCGTTACAGCA-3′, murine *VEGFA* probe (FAM), 5′-CCGCTGATGCGCTGTGCAGG-3′; murine *VEGFC* forward, 5′-AAGACCGTGTGCGAATCGA-3′; murine *VEGFC* reverse, 5′-ACACAGCGGCATACTTCTTCAC-3′; murine *VEGFC* probe (FAM), 5′-TGAAGCATTGTGATCCAGGACTGTCCTTT-3′; murine *TGFbeta* forward, 5′-AAACGGAAGCGCATCGAA-3′; murine *TGFbeta* reverse, 5′-GGGACTGGCGAGCTTAGTT-3′; murine *TGFbeta* probe (FAM), 5′-CCATCCGTGGCCAGATCCTGTCC-3′; murine *Ym1* forward, 5′-CATTGGAGGATGGAAGTTTGGA-3′; murine *Ym1* reverse, 5′-GAATATCTGACGGTTCTGAGGAGTAGA-3′; murine *Ym1* probe (FAM), 5′-CTGCCCCGTTCAGTGCCATGGT-3′. Human and murine *GAPDH* primers and probe sets (VIC) were obtained from Applied Biosystems. Real-time PCR reagents were the Taqman Universal Master Mix II (Applied Biosystems) or SYBR Green PCR Master Mix (Applied Biosystems), and all PCR analyses were performed on an ABI Prism 7900HT.

### Histology

Lung tissues were resected, formalin-fixed, and paraffin-embedded using standard methods. Tissue sections were evaluated microscopically for tumor progression indices using H&E staining.

### ELISA

Differentiated macrophages were stimulated with appropriate concentrations of LPS/IFNγ for 12 hrs. Supernatants were tested for the presence of cytokines using a commercially available multiplex ELISA for IL-1β. ELISA was performed for IL-6 (R&D Systems).

### Confocal microscopy and image analysis

A total of 105 human monocytes per chamber were seeded onto Lab-Tek four-chamber slides (Nunc) and allowed to differentiate for 7 days. Macrophages were washed once with PBS and fixed with 4% paraformaldehyde. Chambers were washed 3 times (5 min each) with PBS followed by 300 nM DAPI (Invitrogen) staining for 1 min at room temperature in the dark. Chambers were washed 3 times (5 min each), plastic chamber inserts were removed, and slides were coverslipped with ProLong Gold antifade reagent (Invitrogen). Slides were evaluated and captured with an OLYMPUS FluoView 1000 Confocal Microscope (OLYMPUS). Merged images were created with OLYMPUS FLUOVIEW Viewer Software, version 2.0. Attached macrophages were quantified using Image J software and plotted using GraphPad software, version 5.0.

### Bacterial infection

Human mononocytes (0.5×10^6^) in 500 µl of complete media were differentiated for 7 days on a 24-well plate with or without etodolac treatment. *Staphylococcus aureus* (serotype V) was grown in Luria broth with agitation at 37°C to an OD_600_ of 0.4, which is equivalent to 1×10^8^ cfu/ml. A total 0.2×10^6^ cfu of unwashed *S. aureus* was used to infect each well. Plates were centrifuged for 5 min at 400×*g* and incubated for 1 hr. The resulting supernatants were subjected to serial 10-fold dilution in water prior to dispensing 100 µl of each dilution onto agar plates. The plates were incubated upside down at 37°C, and bacterial colonies were counted 24 hrs later.

### Serum High Performance Liquid Chromatography (HPLC)

HPLC was performed on a Hewlett-Packard 1100 series (Korea Basic Science Institute, Seoul). The etodolac standard and samples were separated on OP-C18 (250×4.6 mm, 5 µm, RStech Corporation) and detected by absorbance at 274 nm. The mobile phase was 0.05% H_3_PO_4_: acetonitrile (1∶1) and the flow rate was 1.0 ml/min. Injection volume was 100 µl. For quantification of etodolac in samples, a linear calibration plot was obtained in the concentration range 0.5–5.0 µM.

### MTT assay

4T1 cells (10^4^/500 µl complete media) were seeded on a 24-well plate and allowed to attach overnight. Etodolac was added at 0, 10, 20, 50, 100, and 500 µM. An MTT assay was performed at each time point (24, 48, or 72 hrs). Fifty microliters MTT (Sigma Aldrich) was added to each well and further incubated for 4 hrs at 37°C with 5% CO_2_. After washing off supernatants, 500 µl of 100% DMSO was added and the plate was shaken for 5 min. The absorbance at 560 nm was measured using a Beckman ELISA reader.

#### Statistical analyses

Student's t-tests were performed to determine statistically significant differences between groups using GraphPad Prism (GraphPad Software, CA, USA). A P-value<0.05 was considered significant.

## Results

### Etodolac inhibits human alternatively activated macrophage phenotype

**Figure 1:**
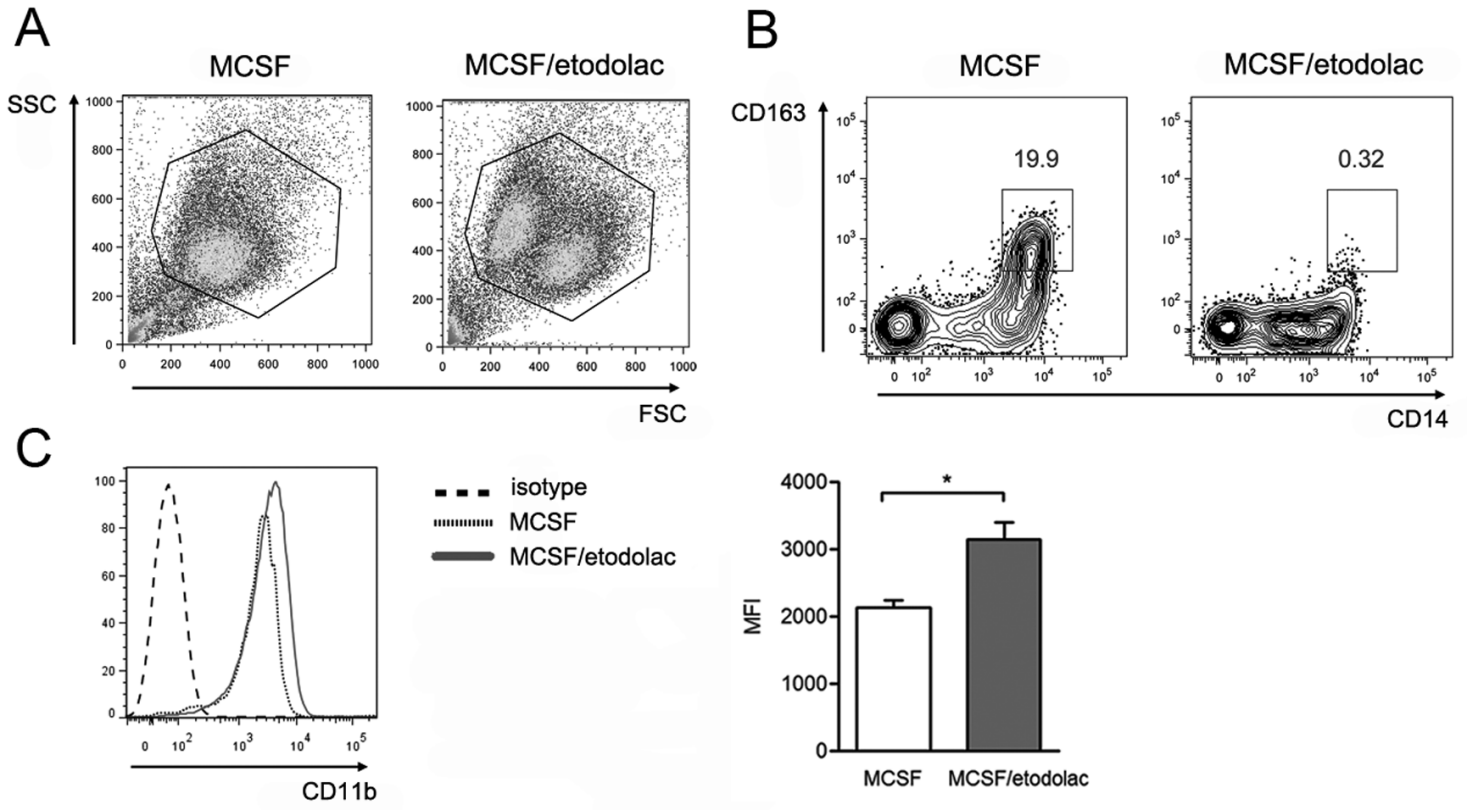
Etodolac inhibits human alternatively activated macrophage phenotype. ***Panel A*** **:** Representative scatter plots of primary human macrophages (solid gates) from five healthy individual donor blood monocytes. Human monocytes were differentiated for 7 days with 20 ng/ml M-CSF in the presence or absence of 20 µM etodolac. ***Panel B*** **:** Dot plots indicating the CD14 and CD163 double positive M2 macrophage population (inbox). Representative of five experiments. ***Panel C*** **:** Histograms (left) and quantitative MFI graphs (left) for CD11b. Error bars, SEM, *, *p*<0.05. Representative of three experiments.

To mimic the tumor microenvironment in vitro culture conditions, we used M-CSF as a tumor-associated, macrophage-assisted growth factor. Normal human peripheral blood monocytes were isolated using magnetic bead negative selection. After differentiation for 7 days with 20 ng/ml M-CSF in the presence or absence of etodolac, macrophages were examined for surface marker expressions (**Fig. 1**). Scatter plots showed that etodolac-treated macrophages have distinct subpopulations compared to those treated with M-CSF alone (**Fig. 1A**). M-CSF-induced macrophages had CD14/CD163 double positive population which represents M2 polarization (**Fig. 1B**) but etodolac abolished CD163 induction almost completely, and partially abolished CD14 induction. Etodolac also increased CD11b expression (**Fig. 1C**). As shown in **Figure S1A and S1B**, GM-CSF-induced classically activated macrophages from human monocytes do not have CD14/CD163 double positive population but had higher CD11b expression compared to M-CSF-induced M2 macrophages [29]. We supposed from these results that COX-2 activity might be required for M2 macrophage differentiation. Other macrophage characteristics were also examined. Macrophage morphology often represents their activation state, and we confirmed that etolodac induced different macrophage morphologies (**Fig. S1C**). M-CSF-treated macrophages at 20 ng/ml concentration exhibited predominantly a smaller and crumpled morphology whereas etodolac-treated macrophages were larger and elongated. Etodolac increased macrophage attaching ability but reduced macrophage phagocytic ability upon *Staphylococcus aureus* infection (**Fig. S1D**), indicating reduced scavenger receptor expression, which is associated with the classically activated macrophages [28]. Collectively, these in vitro data suggest that etodolac altered human M2 macrophage differentiation process and that COX-2 might be required to normal alternatively activated macrophage development.

### Etodolac induces pro-inflammatory cytokines but inhibits pro-metastatic molecules in human macrophages

**Figure 2:**
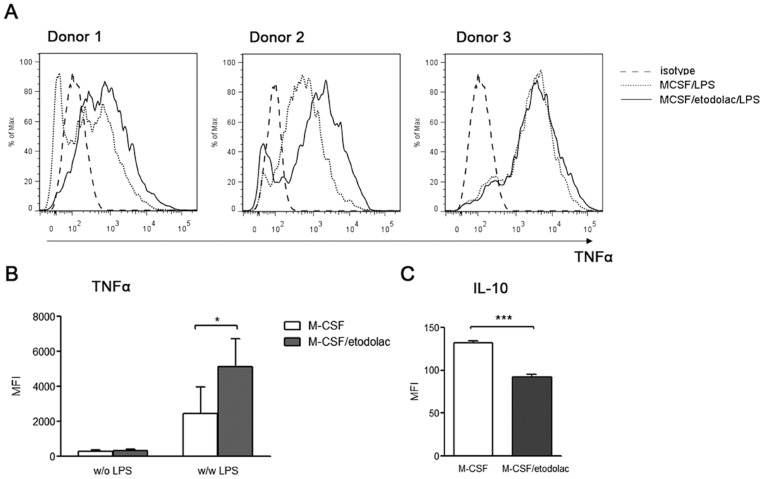
Etodolac induces TNFα but reduces IL-10 of human macrophages. ***Panel A:*** Histograms for intracellular TNFα measured by FACS. Human monocytes from three independent donors were differentiated during 7 days with of without 20 µM etodolac. Quantitative MFI graphs for intracellular TNFα **(** ***Panel B*** **)** and IL-10 **(** ***Panel C*** **)** measured by FACS. LPS stimulation during 5 hrs before examination. Error bars, SEM, *, *p*<0.05, **, *p*<0.01 by Student's *t*-test.

**Figure 3:**
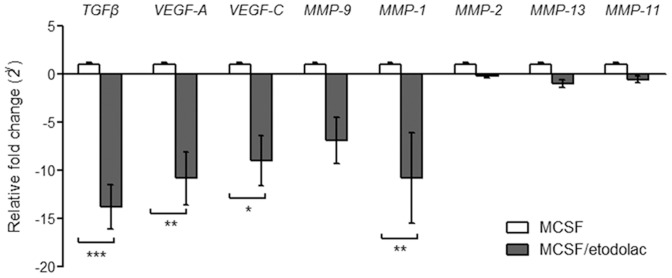
Etodolac reduces pro-metastatic genes in human macrophages. Real-time PCR analysis of *in vitro*-differentiated human macrophages. A total of 50 ng cDNA was analyzed by each primer/probe set. Data represents relative fold changes of etodolac-treated macrophages compared with control, after normalization to an endogenous *GAPDH* control. Error bars, SEM, *, *p*<0.05, **, *p*<0.01, ***, *p*<0.001 using two-way ANOVA with Bonferroni correction, *n* = 3.

Classically activated macrophages induce rapid inflammation, mediated mainly by various early cytokines, such as TNFα, IL-1β, and IL-6 upon stimuli. In contrast, alternatively activated macrophages secrete IL-10 or TGFβ and contribute to tissue homeostasis. To determine whether etodolac-treated macrophages exhibit a different cytokine panel, we first examined intracellular TNFα accumulation during 5 hrs upon LPS stimulation (**Fig. 2A**) using FACS. As expected, macrophages produced increased TNFα in the presence of etodolac. Basal IL-10 synthesis levels were lower in the etodolac treated macrophages. IL-1β and IL-6 in the culture supernatant of M-CSF/etodolac-treated macrophages after LPS/INFγ stimulation also increased than control (**Fig. S1E**). In addition to cytokines, several tumor metastasis-related genes showed the same tendency. Etodolac reduced the gene expression levels of *TGFβ*, *VEGFA*, *VEGFC*, *MMP-9*, and *MMP-1* in macrophages (**Fig. 3**). Taken together, these data further indicate that COX-2 is required for normal M2 or tumor-associated macrophage differentiation.

### Etodolac induced more immune activated murine macrophages

**Figure 4:**
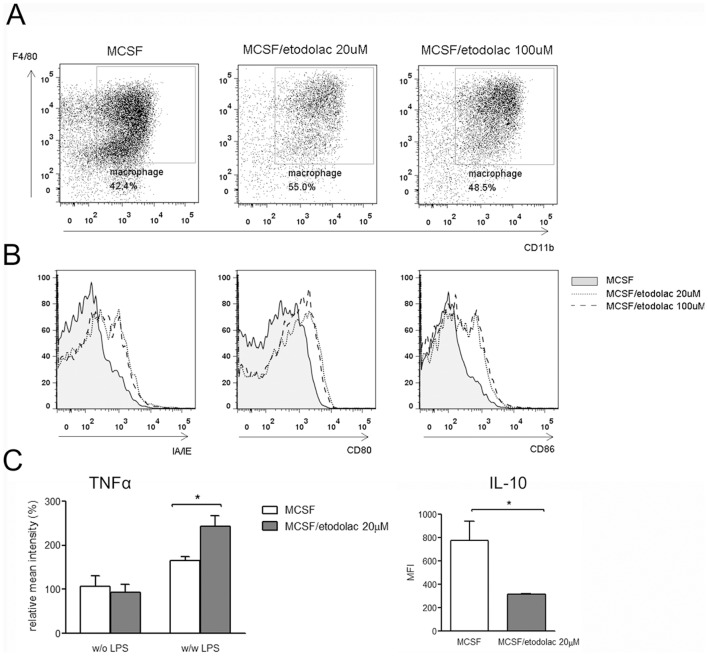
Etodolac induced more immune activated murine macrophages. ***Panel A*** **:** BALB/c bone marrow cells were differentiated with 10% L929 culture supernatants during 7 days and CD11b F4/80 double positive macrophage populations were backgated for further examinations by FACS. Etodolac 20 µM or 100 µM was applied from the start of culture and until examination. ***Panel B*** **:** Merged histograms represents macrophage surface IA/IE, CD80 and CD86 expressions. Representatives for five independent experiments. ***Panel C*** **:** MFI for intracellular TNFα and IL-10 measured by FACS. Golgitransporter inhibitor was applied during 5 hrs with or without 100 ng/ml LPS stimulation. Error bars, SEM, *, *p*<0.05 by Student's *t*-test.

To determine if these effects of COX-2 inhibition are also applied in murine macrophages, BALB/c bone marrow derived macrophages were tested for their markers and cytokine expressions. M-CSF secreting cell line L929 culture supernatants were supplemented in 10% as tumor microenvironmental monocyte differentiating factors, and these condition gives about 50% of CD11b F4/80 double positive mature macrophage population (**Fig. 4A**). Etodolac induced more MHCII IA/IE and co-receptors CD80 and CD86 on macrophage surfaces both in 20 and 100 µM (**Fig. 4B**). In the case of cytokines, TNFα expression levels were higher but IL-10 was lower in etodolac treated macrophages. These results showed that COX-2 inhibition also reduced murine M2 macrophage function.

### Etodolac inhibits lung metastasis in a murine syngeneic breast cancer model

**Figure 5:**
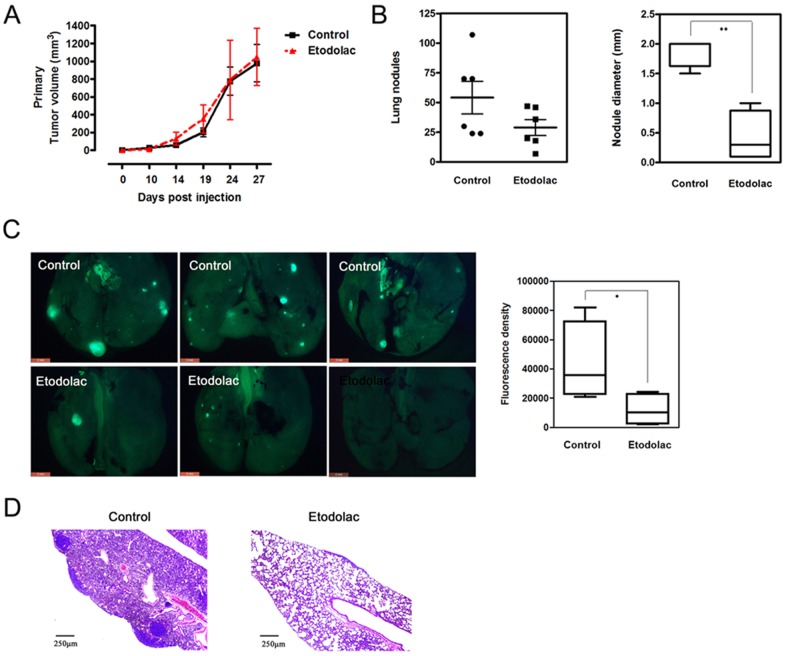
Etodolac inhibits lung metastasis in a murine syngeneic breast cancer model. *****Panel A*:**** Primary tumor volumes were measured at the indicated time point. Feeding with 500 ppm etodolac-containing food started 7 days before tumor cell injection (10^5^ cells) into the right inguinal mammary fat pad of 7-week-old female BALB/c mice and continued until sacrifice. n = 6. ***Panel B*** **:** Metastatic lung nodules on lung surfaces were manually counted and measured in size after resecting lungs from sacrificed mice. Means of a total of three separate counts. *p*<0.05, Mann-whitney U test, n = 6. ***Panel C*** **:** Lung metastasis images obtained by fluorescence microscopy after 4T1_GFP cell implantation (Left panel). Right graph represents quantitatively analyzed fluorescence density measured using Image J software (*p* = 0.0377, Student's unpaired *t*-test, *n* = 3). ***Panel D*** **:** Lung histology by HE staining. Scale bars, left bottom.

Next, to examine the effects of etodolac on tumor progression, we used a murine syngeneic breast cancer model. Female BALB/c mice (age 6 weeks) were fed food containing 500 ppm etodolac, starting one week before injecting with 105 4T1 murine breast tumor cells in the right mammary fat pad until the final sacrifice date. We did not observe any side effects of etodolac treatments during the experimental periods, as represented by mouse feeding and weight data (**Fig. S2A**). There have been several reports regarding direct COX-2 mediated tumorigenesis [22], [30], but we did not observe any COX-2-mediated cellular growth advantage in the 4T1 cell line (**Fig. S2B**). Consistent with these data, primary tumor volumes were not different between control and etodolac-treated mice (**Fig. 5A**). However, metastatic lung nodules were significantly lower both in the number and size in etodolac treated group (**Fig. 5B**). 4T1_GFP cell implantation further confirmed that lung metastatic nodules of etodolac treated mice are fewer in number and smaller in size (**Fig. 5C**). H&E staining showed that the etodolac-treated group had fewer and smaller lung nodules than the non-treated group (**Fig. 5D**). We infered from these results that tumor cell metastasis may be inhibited due to COX-2 inhibition during macrophage differentiation.

### Etodolac induced more immune activated macrophages in breast tumor

**Figure 6:**
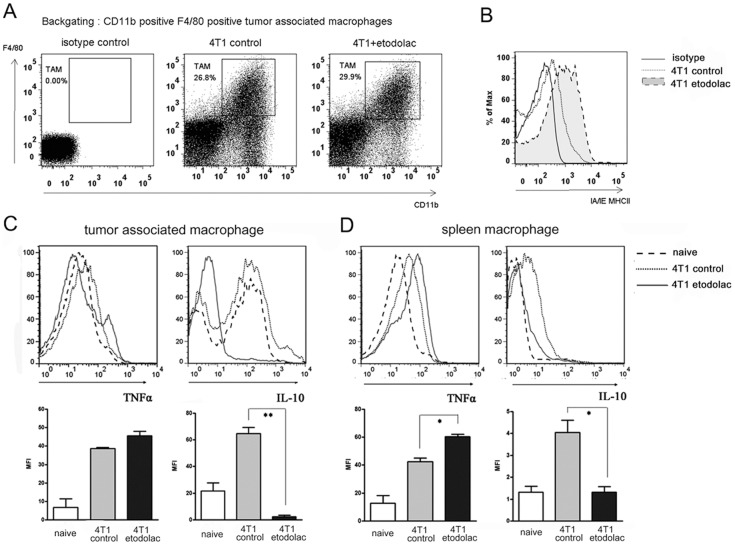
Etodolac induced more immune activated macrophages in breast cancer. ***Panel A*** **:** Dot plots from primary breast tumor single cell isolates. Inset gate, CD11b F4/80 double positive macrophages as backgating using FACS. ***Panel B*** **:** Merged histograms for IA/IE expression on macrophage surfaces by FACS. Representative for five animals per each group. ***Panel C*** **:** Merged histograms for intracellular TNFα (top left) and IL-10 (top right), all gated on F4/80^+^CD11b^+^ macrophages from naïve peritoneal fluid cells and primary breast tumors. Bottom graphs represents quantitative MFI. n = 3, **, *p*<0.01 by student *t*-test. ***Panel D*** **:** Merged histograms for intracellular TNFα (top left) and IL-10 (top right), all gated on F4/80^+^CD11b^+^ macrophages from naïve and tumor implanted splenocytes. Bottom graphs represents quantitative MFI. n = 3, *, *p*<0.05 by student *t*-test.

**Figure 7:**
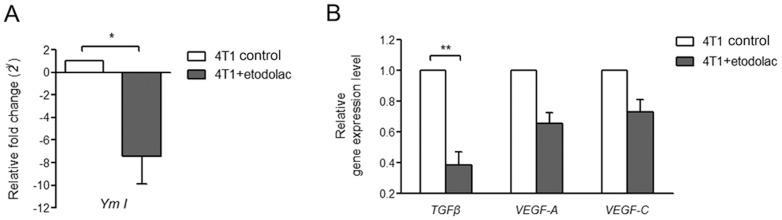
Etodolac inhibited *YmI* and *TGFβ* of TAMs in breast cancer. *YmI* ***(Panel A)*** and *TGFβ*, *VEGFA* and *VEGFC* ***(Panel B)*** gene expression levels were quantified by real-time PCR. Macrophages were obtained from primary breast tumors using CD11b-positive magnetic bead selection after single cell digestion followed by RNA extraction. Macrophages were quantified using the relative C_T_ method after endogenous *GAPDH* normalization. *, *p*<0.05, unpaired Student's *t* test.

During tumor progression, tumor cells build up an immunosuppressive microenvironment in which TAMs [27], mostly M2 macrophages, promote angiogenesis and metastasis. To examine if etodolac change immune status of TAMs, we investigated TAMs and spleen macrophages for receptor expression and cytokine contents. Primary tumors had large CD11b F4/80 double positive macrophage population, up to 25∼30% (**Fig. 6A**). In accordance with in vitro results, TAMs showed higher levels of MHCII IA/IE molecules in etodolac-treated mice (**Fig. 6B**), which correlated with increased macrophage immune activities [31]. TAMs contained less intracellular IL-10 (**Fig. 6C**), and splenic macrophages had more TNFα but less IL-10 due to etodolac (**Fig. 6D**). Next we isolated F4/80 positive macrophages and examined the expression levels of genes related to tumor metastasis. Etodolac-treated spleen macrophages exhibited reduced *Ym I* gene expression, a murine M2 macrophage marker (**Fig. 7A**). Etodolac-treated TAMs had decreased levels of *TGFβ*, *VEGFA*, and *VEGFC* mRNA (**Fig. 7B**). TGFβ is well-known as a major immunosuppressive cytokine produced by alternatively activated macrophages and enhances tumor cell epithelial-to-mesenchymal transition, followed by metastasis [32]. VEGFA recruits angiogenic endothelial precursors, and VEGFC guides lymphangiogenesis [33]. Taken together, these data imply that etodolac may reduce M2 macrophage polarization of TAMs and thus inhibit breast tumors to lung metastasis.

## Discussion

Macrophages are a prominent component of the tumor stroma and infiltrate into human and experimental mouse tumors [7], [15], [34]–[38]. In the relationship between macrophages and tumor cells, several molecules, including cytokines, growth factors, and proteolytic enzymes, are known to be essential for successful tumor cell invasion [3], [34], [37], [39]. Accumulating data regarding macrophage polarization indicate the existence of opposite-characterized macrophage subsets known as anti-tumoral classically activated M1 macrophages and pro-tumoral alternatively activated M2 macrophages. TAMs have much in common with M2 macrophages because they promote angiogenesis through VEGF, enhance macrophage recruitment by M-CSF or TGFβ secretion, and promote local invasion through cathepsin and metalloproteinases [34]. Moreover, tumor cell dispersion during early-stage macrophage migration may be linked to recurrence post-surgery [25]. The exact mechanisms of macrophage polarization need to be elucidated to determine their therapeutic potential and ability to pharmacologically control macrophage infiltration in tumors.

Macrophage polarization can be represented experimentally using M-CSF and GM-CSF. M-CSF-induced monocyte differentiation produces mature M2 type macrophages both in human and mice. Human M2 macrophages express high levels of the surface scavenger receptor CD163 and GPI-coupled receptor CD14 but lower levels of CD11b compared with M1 macrophages. Murine M2 macrophage markers includes *YmI*, *FizzI* and *ArginaseI* as well as several surface markers including CD206 and CD209. Both human and murine M1 macrophages express iNOS and IL-12 gene expressions. In our in vitro experimental systems, COX-2 inhibition did not reduce all of above M2 markers but partially changed several markers including CD163 (human), CD11b (human), and *YmI* (murine). Actually, *IL-12* gene expression is the hallmark of M1 polarization, and COX-2 pathway does not seemed to regulate *IL-12* under M2 differentiation signals (data not shown). And transient COX-2 blocking in already matured macrophages did not convert their phenotype into M1 type and only COX-2 inhibition during whole differentiation period showed the above polarizing effects. Thus we conclude that COX-2 signaling pathway during differentiation is one of required prerequisites for alternatively activated macrophage development and COX-2 blocking during differentiation could make some leakage in fully charged M2 macrophages.

Human M2 macrophages differentiated with M-CSF appear crumpled and relatively slim, slightly attached to the flask, or float individually. These morphologies specifically extremes when human monocytes were co-cultured with several cancer cell lines including MDA-MB-231, HT29, MeWo (data not shown). Functionally, they secrete lower levels of various inflammatory cytokines such as TNFα, IL-1β, IL-12, and IL-6 in response to LPS stimulation, but secrete more TGFβ and IL-10 than do M1 macrophages. In contrast, GM-CSF induced M1 macrophages firmly attach to the flask, broaden their cytoplasm, and express low CD14 and CD163 but high CD11b (**Fig. S1A** and **S1B**). They capture and phagocytose less bacteria than do M-CSF-oriented M2 macrophages (**Fig. S1D**) but secrete increased amounts of pro-inflammatory cytokines and chemokines. Based on these immune enhancing effects of classically activated macrophages, the GVAX cancer vaccine platform has been successful in several clinical trials involving administration of GM-CSF-secreting autologous tumor cells in that those results indicated enhanced anti-tumor immunity [40]. In this study, we found that COX-2 inhibition during M-CSF induced macrophage differentiation resulted in effects somewhat similar to those of GM-CSF. This indicates that during monocyte differentiation, COX-2 is the key enzyme for M2 polarization and that blocking this enzyme may be used as a therapy for induction of enhanced anti-tumor immunity.

The exact signaling pathways involved in M2 polarization remain largely unknown. The classic pathway involves the M2-priming cytokines IL-4 and IL-13 inducing STAT6 phosphorylation [41]. Recently, the PI3K/Akt pathway was identified as an M2-inducing signaling pathway conferred by serum amyloid P [42]. IL-4 induces the production of PPAR ligands in macrophages by induction of 12/15 lipoxygenase followed by lipoxin A4 (LXA4) [43]. We confirmed STAT3 phosphorylation and COX-2 induction in M-CSF-treated macrophages (data not shown). No COX-2 band was detected, but increased levels of activated STAT3 were detected in etodolac-treated macrophages. Further study of COX-2 inhibition and STAT3 activation during macrophage polarization is necessary. Meanwhile, COX-2 induces PGE_2_ at the early inflammatory stage, which has been suggested to induce angiogenesis. We attempted to examine the relationship between PGE_2_ and macrophage polarization using protein kinase C and cAMP activator, but did not observe PGE_2_-mediated intracellular signaling effects on macrophage phenotype. However, 15d-PGJ_2_ production during monocyte differentiation was interesting because it increased and peaked on day 5 of differentiation under M-CSF and was completely inhibited in etodolac-treated macrophages (**Fig. S3**). 15d-PGJ_2_ has been shown to possess anti-inflammatory properties, which are conferred via different mechanisms including activation of the prostaglandin D2 receptor 2 (DP2), peroxisome proliferator activated receptor-γ (PPAR-γ), and covalent modification of cysteine thiols in target proteins, such as IκB kinase β (IKKβ) and p65 of NF-κB [44], [45]. Derek et al. also reported that inducible cyclooxygenase may have anti-inflammatory properties partially via 15d-PGJ_2_ [46]. It will be interesting to determine whether 15d-PGJ_2_ has the potential to differentiate monocytes into alternatively activated macrophages or just primes mature macrophages into the M2 type.

Continuous NSAID intake did not influence 4T1 cell proliferation in a BALB/c breast cancer model, but inhibited lung metastasis. TAMs and splenic macrophages showed higher MHCII and pro-inflammatory TNFα expression, indicating that systemic innate immunity was enhanced by etodolac intake. Actually, we observed macrophage enhancing activity even after one week of etodolac feeding before tumor cell injection (**Fig. S4**) in that these mice had more activated peritoneal macrophages and adipose tissue macrophages and consequently produced more serum TNFα and IL-12 against LPS challenge. Our results are in accordance with recently published article [47] in part, which shows COX-2 inhibition alters the phenotype of tumor-associated macrophages from M2 to M1 in *Apc^Min/+^* mouse polyps. The author clarified the effect of celecoxib intake mainly on primary polyp size together with whole tumor cytokine mileu. Our study further extent these phenomenon more precisely showing individual TAM's characteristics in mouse breast cancer model, as well as in human monocyte derived macrophages. COX-2 inhibition potentiates macrophage's inflammatory cytokine responses but reduced IL-10 secretion thus might skew overall tumor microenvironment to favor Th1 immune responses which was confirmed by reduced regulatory T cells as well as myeloid derived suppressor cells in etodolac fed mice tumor mass (data not shown). Up to our knowledge, this is the first describes about NSAIDs mediated human macrophage polarization. Reducing M2 macrophage characteristics systemically may provide advantages to cancer patients because they express much lower levels of *TGFβ*, *VEGFA*, *VEGFC*, *MMP-9*, and *MMP-1* (**Fig. 3**). All of these molecules enhance tumor cell metastasis directly or indirectly [25]. This is significant because most cancer patients who undergo resection surgery have recurrences. Alternatively activated macrophages, which actively secrete the above molecules, could generate single circulating tumor cells before surgery, preventing tumor relapse. Targeting COX-2 and macrophage polarization may help reduce the rate of relapse.

We report that COX-2 inhibition reduces lung metastasis in an experimentally induced breast cancer model. Because COX-2 is an enzyme for differentiation of monocytes into alternatively activated macrophages, COX-2 inhibition may inhibit acquisition of tumor-promoting characteristics of TAMs, including immune-suppressive cytokine secretion, growth factor synthesis, as well as abundant metalloproteinase secretion into the tumor microenvironment. Successive adjuvant therapies targeting VEGF, CD47, CD40, or immune-inhibitory molecules such as PD-1 and CTLA-1, in combination with immune enhancing cytokines, are now considered clinically promising. The majority of these approaches are directly or indirectly associated with macrophage functions because macrophages are the harmful population in the tumor microenvironment. In conclusion, our study provides valuable insights regarding TAM regulation that will facilitate prevention of tumor metastasis.

## Supporting Information

Figure S1 **Human macrophage phenotypes with COX-2 inhibition.** ***Panel A*** **:** Human monocytes were differentiated into M1 under GM-CSF, or M2 under M-CSF during 7 days. Surface CD14 and CD163 expressions were examined using FACs. ***Panel B*** **:** Merged histograms for CD11b expression was obtained by FACS. Representative for three experiments. ***Panel C*** **:** Confocal DIC and DAPI merged images (left upper three, bar = 10 µm) and DAPI stained nucleus (left bottom three, bar = 50 µm) of attached macrophages. Differentiated macrophages after 7 days with M-CSF or M-CSF/etodolac were fixed on four-well chamber slides. Attached cells were quantified (right graph) using Image J software from DAPI images at 40×magnification. Data from three independent experiments, ***, *p*<0.001 by unpaired *t*-test. ***Panel D*** **:** Colonies indicating remnant *Staphylococcus aureus* in the culture supernatant after macrophage phagocytosis during (left image). Monocytes (0.5×10^6^) were differentiated in a 24-well plate. Infection was performed with 0.2×10^6^ cfu *Staphylococcus aureus* for 1 hr after 4 and 7 days of differentiation. A 1∶100 dilution of 100 µl culture supernatants was cultured on LB agar plates and colonies were counted manually (right graph). A total of two independent experiments were performed. *, *p*<0.05 by Student's *t*-test. ***Panel E*** **:** Human IL-1β and IL-6 levels in macrophage culture supernatants were measured using ELISA. Human monocytes from five independent healthy donors were differentiated *in vitro* for 7 days. On the sixth day of differentiation, LPS/IFNγ (100 ng/ml, 25 ng/ml) was added to each group and incubated for another 24 hrs. **, *p*<0.01 by unpaired Student's *t*-test.
(TIF)

Figure S2 **Etodolac did not show any toxicity both in mice and 4T1 cells**. Food consumed ***(Panel A)*** and mouse weight ***(Panel B)*** were measured at the indicated time points. Data are represented as mean grams of consumed feed per mouse per day (*n* = 6). ***Panel C*** **:** MTT assay of etodolac cytotoxicity in the 4T1 cell line. A total of 10^4^ cells/well (in 24-well plates) in 500 µl complete RPMI media were treated with 0–100 µM etodolac. Experiments were repeated three times.
(TIF)

Figure S3 **Etodolac inhibitied 15d-PGJ_2_ production during M2 macrophage differentiation.** 15d-PGJ_2_ ELISA of human primary macrophage cellular protein extracts ***(Panel A)*** and culture supernatants ***(Panel B)*** differentiated with 20 ng/ml M-CSF in the presence or absence of 20 µM etodolac. Data shown are means with SEM of three independent experiments.
(TIF)

Figure S4 **Etodolac intake enhances systemic innate immune responses in BALB/c mice.** ***Panel A*** **:** Six weeks of BALB/c female mouse was fed 500 ppm etodolac containing food during seven days and examined surface MHCII (IA/IE) expressions of peritoneal macrophages using FACS. Upper dot plots represents peritoneal macrophage gating strategies (CD45^+^CD11b^+^F4/80^+^) and lower histogram and bar graph shows enhanced IA/IE expressions. *, *p*<0.05 by unpaired Student's *t*-test. n = 5. ***Panel B*** **:** Adipose tissues were examined for their M2 markers *Arginase I*, *Fizz I*, and *Ym I* gene expressions by real-time PCR. Adipose tissues from etodolac fed mice had reduced M2 marker expressions. **, *p*<0.01 by unpaired student's *t*-test. n = 3. ***Panel C*** **:** Mice were injected with 50 µg of LPS intraperitoneally and blood was collected one hour after injection. Serum TNFα and IL-12 were detected by ELISA. **, *p*<0.01 by unpaired Student's *t*-test. n = 6.
(TIF)

## References

[1] Almand B, Clark JI, Nikitina E, van Beynen J, English NR, et al (2001) Increased production of immature myeloid cells in cancer patients: a mechanism of immunosuppression in cancer. J Immunol 166: 678–689. 11123353 10.4049/jimmunol.166.1.678

[2] Lewis CE, Pollard JW (2006) Distinct role of macrophages in different tumor microenvironments. Cancer Res 66: 605–612. 16423985 10.1158/0008-5472.CAN-05-4005

[3] Mantovani A, Sica A (2010) Macrophages, innate immunity and cancer: balance, tolerance, and diversity. Curr Opin Immunol 22: 231–237. 20144856 10.1016/j.coi.2010.01.009

[4] Bingle L, Brown NJ, Lewis CE (2002) The role of tumour-associated macrophages in tumour progression: implications for new anticancer therapies. J Pathol 196: 254–265. 11857487 10.1002/path.1027

[5] Pollard JW (2008) Macrophages define the invasive microenvironment in breast cancer. J Leukoc Biol 84: 623–630. 18467655 10.1189/jlb.1107762 PMC2516896

[6] Sica A, Larghi P, Mancino A, Rubino L, Porta C, et al (2008) Macrophage polarization in tumour progression. Semin Cancer Biol 18: 349–355. 18467122 10.1016/j.semcancer.2008.03.004

[7] DeNardo DG, Andreu P, Coussens LM (2010) Interactions between lymphocytes and myeloid cells regulate pro- versus anti-tumor immunity. Cancer Metastasis Rev 29: 309–316. 20405169 10.1007/s10555-010-9223-6 PMC2865635

[8] Lin EY, Nguyen AV, Russell RG, Pollard JW (2001) Colony-stimulating factor 1 promotes progression of mammary tumors to malignancy. J Exp Med 193: 727–740. 11257139 10.1084/jem.193.6.727 PMC2193412

[9] Meng Y, Beckett MA, Liang H, Mauceri HJ, van Rooijen N, et al (2010) Blockade of tumor necrosis factor alpha signaling in tumor-associated macrophages as a radiosensitizing strategy. Cancer Res 70: 1534–1543. 20145121 10.1158/0008-5472.CAN-09-2995 PMC8849568

[10] Qian B, Deng Y, Im JH, Muschel RJ, Zou Y, et al (2009) A distinct macrophage population mediates metastatic breast cancer cell extravasation, establishment and growth. PLoS One 4: e6562. 19668347 10.1371/journal.pone.0006562 PMC2721818

[11] Gordon S, Taylor PR (2005) Monocyte and macrophage heterogeneity. Nat Rev Immunol 5: 953–964. 16322748 10.1038/nri1733

[12] Mantovani A, Sica A, Locati M (2005) Macrophage polarization comes of age. Immunity 23: 344–346. 16226499 10.1016/j.immuni.2005.10.001

[13] Mantovani A, Sozzani S, Locati M, Allavena P, Sica A (2002) Macrophage polarization: tumor-associated macrophages as a paradigm for polarized M2 mononuclear phagocytes. Trends Immunol 23: 549–555. 12401408 10.1016/s1471-4906(02)02302-5

[14] Talmadge JE, Donkor M, Scholar E (2007) Inflammatory cell infiltration of tumors: Jekyll or Hyde. Cancer Metastasis Rev 26: 373–400. 17717638 10.1007/s10555-007-9072-0

[15] Mantovani A, Allavena P, Sica A, Balkwill F (2008) Cancer-related inflammation. Nature 454: 436–444. 18650914 10.1038/nature07205

[16] Murdoch C, Tazzyman S, Webster S, Lewis CE (2007) Expression of Tie-2 by human monocytes and their responses to angiopoietin-2. J Immunol 178: 7405–7411. 17513791 10.4049/jimmunol.178.11.7405

[17] Ghassabeh GH, De Baetselier P, Brys L, Noel W, Van Ginderachter JA, et al (2006) Identification of a common gene signature for type II cytokine-associated myeloid cells elicited in vivo in different pathologic conditions. Blood 108: 575–583. 16556895 10.1182/blood-2005-04-1485

[18] Solinas G, Schiarea S, Liguori M, Fabbri M, Pesce S, et al (2010) Tumor-conditioned macrophages secrete migration-stimulating factor: a new marker for M2-polarization, influencing tumor cell motility. J Immunol 185: 642–652. 20530259 10.4049/jimmunol.1000413

[19] Pollard JW (2004) Tumour-educated macrophages promote tumour progression and metastasis. Nat Rev Cancer 4: 71–78. 14708027 10.1038/nrc1256

[20] Sinha P, Clements VK, Fulton AM, Ostrand-Rosenberg S (2007) Prostaglandin E2 promotes tumor progression by inducing myeloid-derived suppressor cells. Cancer Res 67: 4507–4513. 17483367 10.1158/0008-5472.CAN-06-4174

[21] Thun MJ, Henley SJ, Patrono C (2002) Nonsteroidal anti-inflammatory drugs as anticancer agents: mechanistic, pharmacologic, and clinical issues. J Natl Cancer Inst 94: 252–266. 11854387 10.1093/jnci/94.4.252

[22] Dannenberg AJ, Altorki NK, Boyle JO, Dang C, Howe LR, et al (2001) Cyclo-oxygenase 2: a pharmacological target for the prevention of cancer. Lancet Oncol 2: 544–551. 11905709 10.1016/S1470-2045(01)00488-0

[23] Wang D, Mann JR, DuBois RN (2005) The role of prostaglandins and other eicosanoids in the gastrointestinal tract. Gastroenterology 128: 1445–1461. 15887126 10.1053/j.gastro.2004.09.080

[24] Iwata C, Kano MR, Komuro A, Oka M, Kiyono K, et al (2007) Inhibition of cyclooxygenase-2 suppresses lymph node metastasis via reduction of lymphangiogenesis. Cancer Res 67: 10181–10189. 17974958 10.1158/0008-5472.CAN-07-2366

[25] Guiet R, Van Goethem E, Cougoule C, Balor S, Valette A, et al (2011) The process of macrophage migration promotes matrix metalloproteinase-independent invasion by tumor cells. J Immunol 187: 3806–3814. 21880978 10.4049/jimmunol.1101245 PMC4276309

[26] Kozin SV, Kamoun WS, Huang Y, Dawson MR, Jain RK, et al (2010) Recruitment of myeloid but not endothelial precursor cells facilitates tumor regrowth after local irradiation. Cancer Res 70: 5679–5685. 20631066 10.1158/0008-5472.CAN-09-4446 PMC2918387

[27] Alitalo K, Tammela T, Petrova TV (2005) Lymphangiogenesis in development and human disease. Nature 438: 946–953. 16355212 10.1038/nature04480

[28] Leidi M, Gotti E, Bologna L, Miranda E, Rimoldi M, et al (2009) M2 macrophages phagocytose rituximab-opsonized leukemic targets more efficiently than m1 cells in vitro. J Immunol 182: 4415–4422. 19299742 10.4049/jimmunol.0713732

[29] Raes G, De Baetselier P, Noel W, Beschin A, Brombacher F, et al (2002) Differential expression of FIZZ1 and Ym1 in alternatively versus classically activated macrophages. J Leukoc Biol 71: 597–602. 11927645

[30] Tsujii M, Kawano S, Tsuji S, Sawaoka H, Hori M, et al (1998) Cyclooxygenase regulates angiogenesis induced by colon cancer cells. Cell 93: 705–716. 9630216 10.1016/s0092-8674(00)81433-6

[31] Beatty GL, Chiorean EG, Fishman MP, Saboury B, Teitelbaum UR, et al (2011) CD40 agonists alter tumor stroma and show efficacy against pancreatic carcinoma in mice and humans. Science 331: 1612–1616. 21436454 10.1126/science.1198443 PMC3406187

[32] Massague J (2008) TGFbeta in Cancer. Cell 134: 215–230. 18662538 10.1016/j.cell.2008.07.001 PMC3512574

[33] Achen MG, Stacker SA (2006) Tumor lymphangiogenesis and metastatic spread-new players begin to emerge. Int J Cancer 119: 1755–1760. 16557570 10.1002/ijc.21899

[34] Gocheva V, Wang HW, Gadea BB, Shree T, Hunter KE, et al (2010) IL-4 induces cathepsin protease activity in tumor-associated macrophages to promote cancer growth and invasion. Genes Dev 24: 241–255. 20080943 10.1101/gad.1874010 PMC2811826

[35] Grivennikov SI, Greten FR, Karin M (2010) Immunity, inflammation, and cancer. Cell 140: 883–899. 20303878 10.1016/j.cell.2010.01.025 PMC2866629

[36] Hallam S, Escorcio-Correia M, Soper R, Schultheiss A, Hagemann T (2009) Activated macrophages in the tumour microenvironment-dancing to the tune of TLR and NF-kappaB. J Pathol 219: 143–152. 19662665 10.1002/path.2602 PMC2935674

[37] Joyce JA, Pollard JW (2009) Microenvironmental regulation of metastasis. Nat Rev Cancer 9: 239–252. 19279573 10.1038/nrc2618 PMC3251309

[38] Sinha P, Clements VK, Bunt SK, Albelda SM, Ostrand-Rosenberg S (2007) Cross-talk between myeloid-derived suppressor cells and macrophages subverts tumor immunity toward a type 2 response. J Immunol 179: 977–983. 17617589 10.4049/jimmunol.179.2.977

[39] DeNardo DG, Barreto JB, Andreu P, Vasquez L, Tawfik D, et al (2009) CD4(+) T cells regulate pulmonary metastasis of mammary carcinomas by enhancing protumor properties of macrophages. Cancer Cell 16: 91–102. 19647220 10.1016/j.ccr.2009.06.018 PMC2778576

[40] Borrello IM, Levitsky HI, Stock W, Sher D, Qin L, et al (2009) Granulocyte-macrophage colony-stimulating factor (GM-CSF)-secreting cellular immunotherapy in combination with autologous stem cell transplantation (ASCT) as postremission therapy for acute myeloid leukemia (AML). Blood 114: 1736–1745. 19556425 10.1182/blood-2009-02-205278 PMC2738565

[41] Martinez FO, Helming L, Gordon S (2009) Alternative activation of macrophages: an immunologic functional perspective. Annu Rev Immunol 27: 451–483. 19105661 10.1146/annurev.immunol.021908.132532

[42] Zhang W, Xu W, Xiong S (2011) Macrophage differentiation and polarization via phosphatidylinositol 3-kinase/Akt-ERK signaling pathway conferred by serum amyloid P component. J Immunol 187: 1764–1777. 21753147 10.4049/jimmunol.1002315

[43] Martinez FO, Sica A, Mantovani A, Locati M (2008) Macrophage activation and polarization. Front Biosci 13: 453–461. 17981560 10.2741/2692

[44] Herlong JL, Scott TR (2006) Positioning prostanoids of the D and J series in the immunopathogenic scheme. Immunol Lett 102: 121–131. 16310861 10.1016/j.imlet.2005.10.004

[45] Kim EH, Surh YJ (2006) 15-deoxy-Delta12,14-prostaglandin J2 as a potential endogenous regulator of redox-sensitive transcription factors. Biochem Pharmacol 72: 1516–1528. 16987499 10.1016/j.bcp.2006.07.030

[46] Gilroy DW, Colville-Nash PR, Willis D, Chivers J, Paul-Clark MJ, et al (1999) Inducible cyclooxygenase may have anti-inflammatory properties. Nat Med 5: 698–701. 10371510 10.1038/9550

[47] Nakanishi Y, Nakatsuji M, Seno H, Ishizu S, Akitake-Kawano R, et al (2011) COX-2 inhibition alters the phenotype of tumor-associated macrophages from M2 to M1 in ApcMin/+ mouse polyps. Carcinogenesis 32: 1333–1339. 21730361 10.1093/carcin/bgr128

