# Supplementary figures and images for: Cyclooxygenase-2 Inhibition Blocks M2 Macrophage Differentiation and Suppresses Metastasis in Murine Breast Cancer Model
Source: PLoS One. 2013 May 7;8(5):e63451. doi: 10.1371/journal.pone.0063451 (PMC3646746; doi:10.1371/journal.pone.0063451)

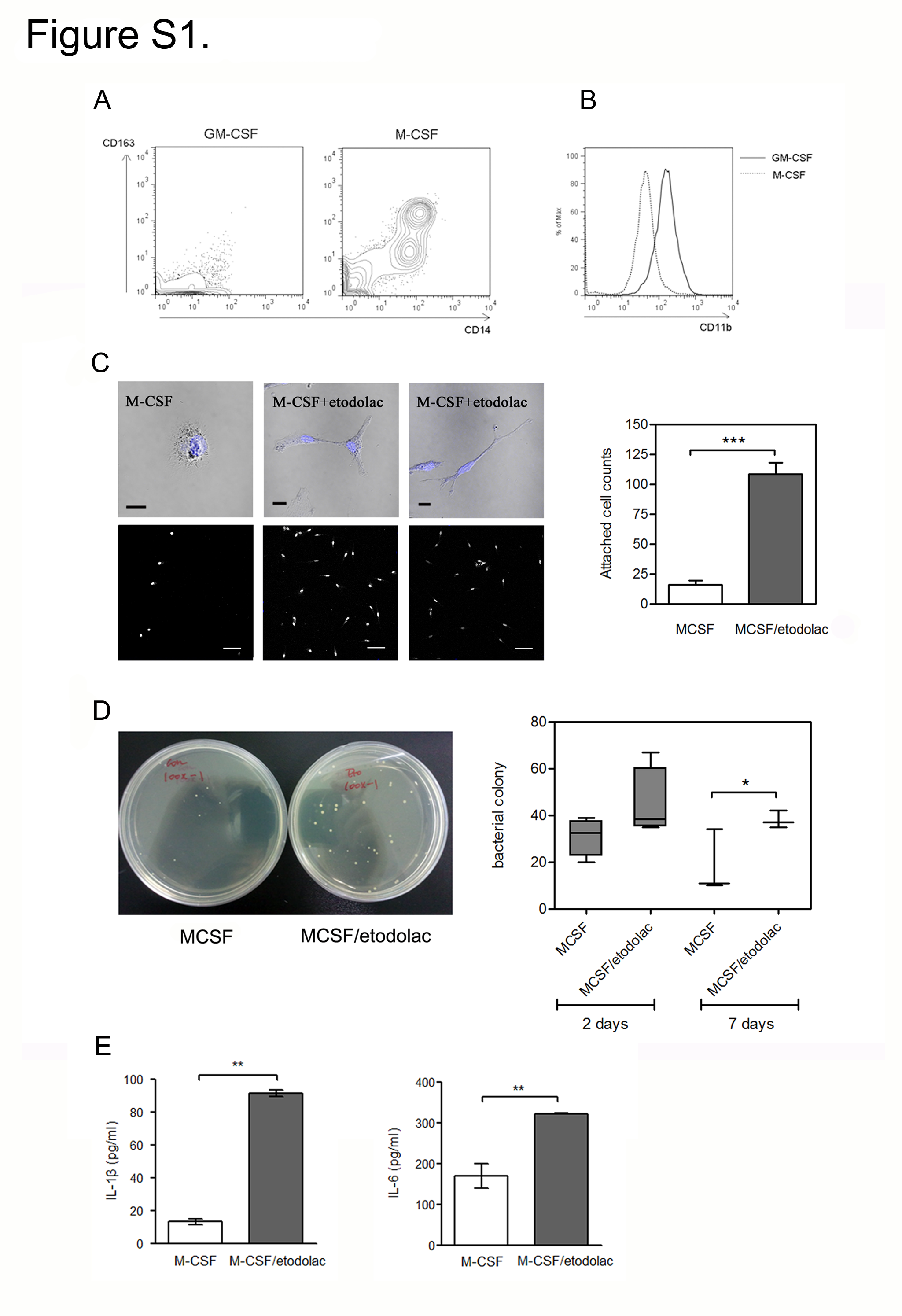

Supplement: Figure S1 — Human macrophage phenotypes with COX-2 inhibition. Panel A : Human monocytes were differentiated into M1 under GM-CSF, or M2 under M-CSF during 7 days. Surface CD14 and CD163 expressions were examined using FACs. Panel B : Merged histograms for CD11b expression was obtained by FACS. Representative for three experiments. Panel C : Confocal DIC and DAPI merged images (left upper three, bar = 10 µm) and DAPI stained nucleus (left bottom three, bar = 50 µm) of attached macrophages. Differentiated macrophages after 7 days with M-CSF or M-CSF/etodolac were fixed on four-well chamber slides. Attached cells were quantified (right graph) using Image J software from DAPI images at 40×magnification. Data from three independent experiments, ***, p<0.001 by unpaired t-test. Panel D : Colonies indicating remnant Staphylococcus aureus in the culture supernatant after macrophage phagocytosis during (left image). Monocytes (0.5×10^6^) were differentiated in a 24-well plate. Infection was performed with 0.2×10^6^ cfu Staphylococcus aureus for 1 hr after 4 and 7 days of differentiation. A 1∶100 dilution of 100 µl culture supernatants was cultured on LB agar plates and colonies were counted manually (right graph). A total of two independent experiments were performed. *, p<0.05 by Student's t-test. Panel E : Human IL-1β and IL-6 levels in macrophage culture supernatants were measured using ELISA. Human monocytes from five independent healthy donors were differentiated in vitro for 7 days. On the sixth day of differentiation, LPS/IFNγ (100 ng/ml, 25 ng/ml) was added to each group and incubated for another 24 hrs. **, p<0.01 by unpaired Student's t-test. (TIF) [file pone.0063451.s001.tif]

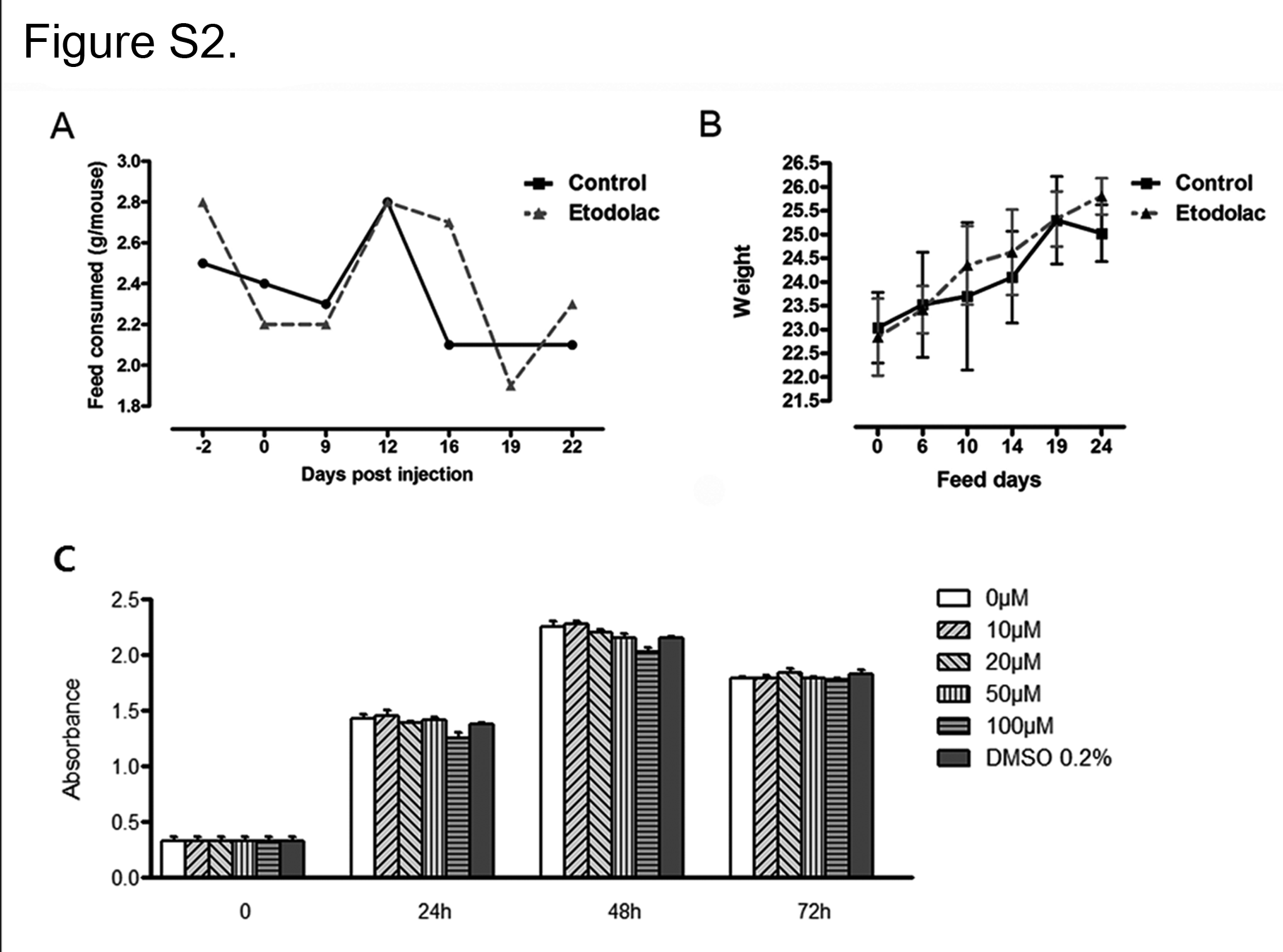

Supplement: Figure S2 — Etodolac did not show any toxicity both in mice and 4T1 cells. Food consumed (Panel A) and mouse weight (Panel B) were measured at the indicated time points. Data are represented as mean grams of consumed feed per mouse per day (n = 6). Panel C : MTT assay of etodolac cytotoxicity in the 4T1 cell line. A total of 104 cells/well (in 24-well plates) in 500 µl complete RPMI media were treated with 0–100 µM etodolac. Experiments were repeated three times. (TIF) [file pone.0063451.s002.tif]

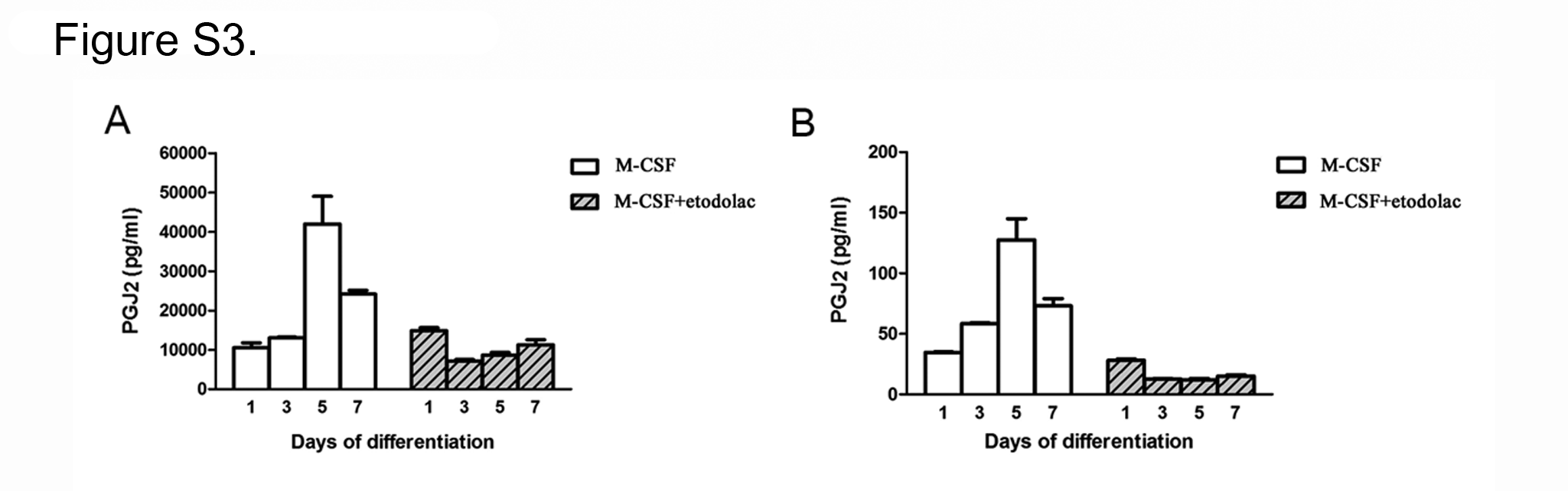

Supplement: Figure S3 — Etodolac inhibitied 15d-PGJ2 production during M2 macrophage differentiation. 15d-PGJ2 ELISA of human primary macrophage cellular protein extracts (Panel A) and culture supernatants (Panel B) differentiated with 20 ng/ml M-CSF in the presence or absence of 20 µM etodolac. Data shown are means with SEM of three independent experiments. (TIF) [file pone.0063451.s003.tif]

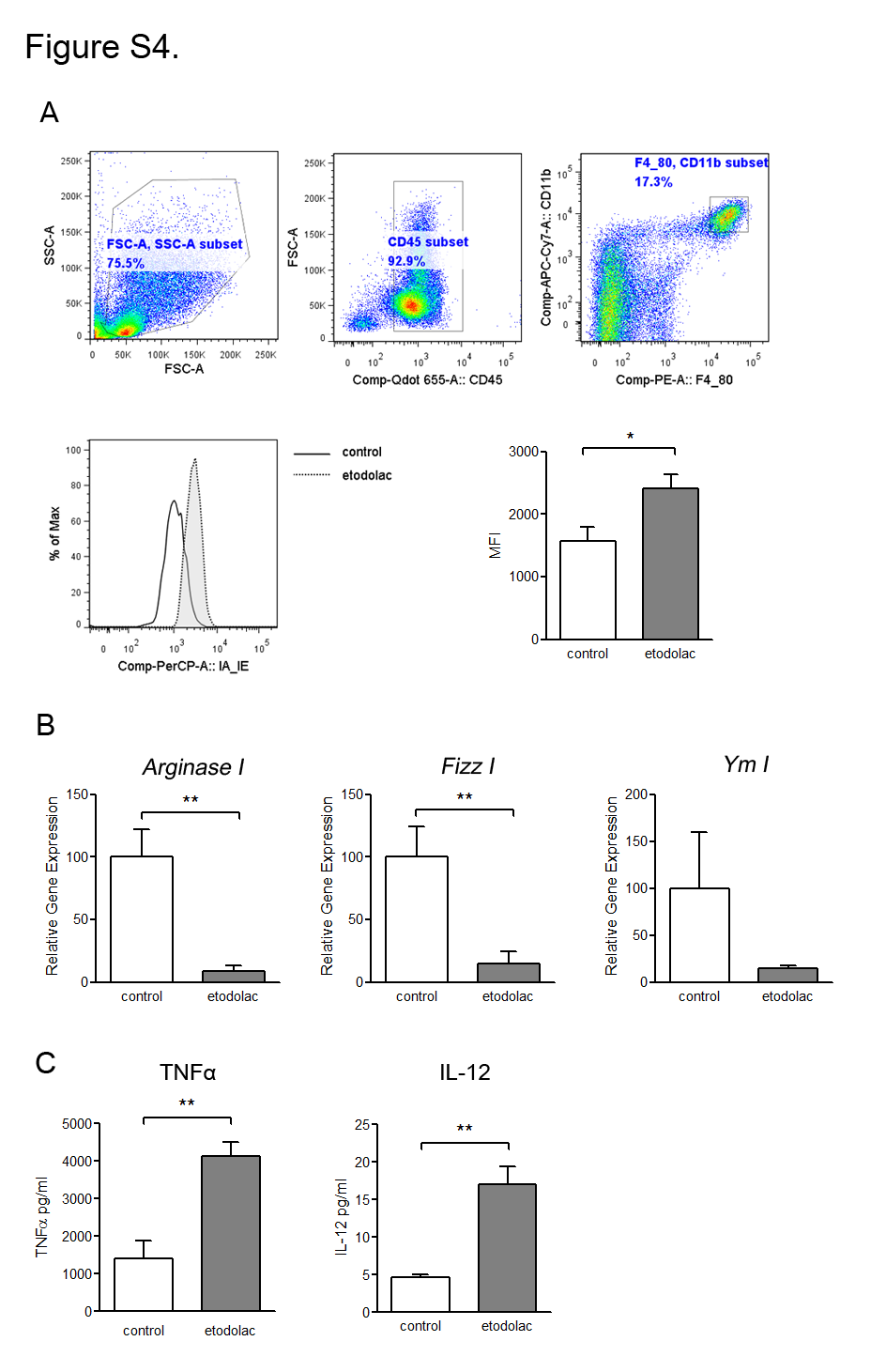

Supplement: Figure S4 — Etodolac intake enhances systemic innate immune responses in BALB/c mice. Panel A : Six weeks of BALB/c female mouse was fed 500 ppm etodolac containing food during seven days and examined surface MHCII (IA/IE) expressions of peritoneal macrophages using FACS. Upper dot plots represents peritoneal macrophage gating strategies (CD45+CD11b+F4/80+) and lower histogram and bar graph shows enhanced IA/IE expressions. *, p<0.05 by unpaired Student's t-test. n = 5. Panel B : Adipose tissues were examined for their M2 markers Arginase I, Fizz I, and Ym I gene expressions by real-time PCR. Adipose tissues from etodolac fed mice had reduced M2 marker expressions. **, p<0.01 by unpaired student's t-test. n = 3. Panel C : Mice were injected with 50 µg of LPS intraperitoneally and blood was collected one hour after injection. Serum TNFα and IL-12 were detected by ELISA. **, p<0.01 by unpaired Student's t-test. n = 6. (TIF) [file pone.0063451.s004.tif]
